# Effect of the Prevalence of HIV/AIDS and the Life Expectancy Rate on Economic Growth in SSA Countries: Difference GMM Approach

**DOI:** 10.5539/gjhs.v8n4p212

**Published:** 2015-08-31

**Authors:** Salisu Ibrahim Waziri, Norashidah Mohamed Nor, Nik Mustapha Raja Abdullah, Peter Adamu

**Affiliations:** 1 Faculty of Economics and Management, Universiti Putra Malaysia, 43400, Serdang, Selangor, Malaysia; 2 Department of Economics, Faculty of Social and Management Sciences, Bauchi State University Gadau (BASUG), Nigeria; 3 Department of Economics, Faculty of Social and Management Sciences, Kaduna State University, Nigeria

## Abstract

The productivity of countries around the globe is adversely affected by the health-related problems of their labour force. This study examined the effect of the prevalence of human immunodeficiency virus/acquired immune deficiency syndrome (HIV/AIDS) and life expectancy on the economic growth of 33 Sub-Saharan African (SSA) countries over a period of 11 years (2002–2012). The study employed a dynamic panel approach as opposed to the static traditional approach utilised in the literature. The dynamic approach became eminent because of the fact that HIV/AIDS is a dynamic variable as its prevalence today depends on the previous years. The result revealed that HIV/AIDS is negatively correlated with economic growth in the region, with a coefficient of 0.014, and significant at the 1% level. That is, a 10% increase in HIV/AIDS prevalence leads to a 0.14% decrease in the GDP of the region. Tackling HIV/AIDS is therefore imperative to the developing Sub-Saharan African region and all hands must be on deck to end the menace globally.

## 1. Introduction

The productivity of many countries around the globe is adversely affected by the health-related problems of their labour force; these include the prevalence of communicable diseases, such as HIV/AIDS. The United Nations (UN) Millennium Declaration in 2000 targeted 189 rich and poor countries (including African countries) to attain improvement in human development by the year 2015. This was followed by the formulation of the Millennium Development Goals (MDGSs), in which 8 targets were set: the eradication of extreme poverty and hunger, achieving universal primary education, promoting gender equality and empowering women, reducing child mortality rates, improving maternal health, combating HIV/AIDS, malaria, and other diseases, ensuring environmental sustainability, and developing a global partnership for development in these countries from 2005 to 2015.

Whilst the countries in the West have attained the MDG goals, many countries in Africa, Asia, and Latin America are still lagging behind in achieving some of the stated goals. Objective one of the Millennium Development Goals is to eradicate extreme poverty and hunger by 2015. None of the African countries has achieved the set target; however, 10 countries are on track to achieve the target: Angola, Benin, Ghana, Liberia, Malawi, Mauritania, Mozambique, Rwanda, Swaziland, and Tanzania (World Bank, 2014). The number of citizens living below $1.25 a day has declined in every region except in SSA. In 1990, the poverty rate in SSA was 51%; although there has been a reduction of 3% in the last 25 years to 48%, it is still above the developing countries’ average (World Bank, 2014). SSA remains the centre of the HIV epidemic, with 70% of all new HIV infections in 2012 occurring in SSA. Nonetheless, the Sustainable Development Goals (SDGs) were launched by the UN on 22 September 2014 during the sixty-ninth UN General Assembly meeting with the purpose of accelerating the achievement of the UN’s mission. The SDGs were introduced to complete the MDGs’ targets beyond 2015. One of the targets of the SDGs is to reduce the annual new infections of HIV/AIDS by three-quarters by 2030 and to ensure universal treatment for HIV/AIDS patients by 2040.

On average, the economic growth of Sub-Saharan Africa is low, and the factors responsible might be observed, while some are obviously unobserved. Okochi and Okpuzor (2011) suggested that the poor economic performance of Sub-Saharan Africa is caused by many factors, amongst which is the prevalence of communicable diseases such as HIV/AIDS. On average, the African health care system is weak, due to a lack of adequate funding, poor infrastructure and scant research to address and manage the growing number of challenges, such as the prevalence of HIV/AIDS and tuberculosis (WHO, 2012).

Some of the empirical literature that highlighted the effect of the prevalence of HIV/AIDS on growth concentrated more on single-country analysis. These studies include: Arndt and Lewis (2000); Asiedu (2012); Bell et al. (2006); Cuddington and Hancock (1995); MacFarlan and Sgherri (2001); and Thurlow and Wobst (2004). Those that focused on cross-country or panel analysis are Asiedu (2012) and Over (1992). The above studies examined the relationships between HIV/AIDS and economic growth using the traditional static panel approach. The current study will contribute to the literature in the following ways. First, a dynamic model is applied as opposed to the traditional static model, because the dependent variable (HIV/AIDS) is dynamic in nature. Second, life expectancy as a proxy for human capital development utilises a more dynamic approach to investigate the health–growth nexus in Sub-Saharan Africa, contrary to literature like Hansen (2013) and Kunze (2014), which utilised static models. This paper is organised in the following sections. Following the introduction, Section 2 provides the theoretical framework, which creates links with our model. Section 3 discusses the review of the literature, while section 4 provides the methodology, which includes the panel model specification and the definition of the variables, data and sources. Section 5 presents the empirical results, comprising a summary of the statistics, difference GMM estimation and a discussion of the findings. Finally, section 6 concludes the research, suggests some policy recommendations and identifies some gaps for future studies to investigate.

## 2. Theoretical Framework

Although numerous theories of economic growth exist in the economic literature, the macroeconomic approach to assessing the effect of health-related issues on economic growth mainly adopts the human capital approach. The stock of health of an individual remains a dominant strategy amongst many health–economic growth studies for measuring well-being, such as the work by Bloom, Canning, and Sevilla (2004), McDonald and Roberts (2006), and, more recently, Mahumud et al. (2013). They consider life expectancy as a proxy for health in measuring economic growth using the production function approach.

This study adopts the renowned Solow’s growth model (1956), assuming a Cobb–Douglas production function with two important factor inputs, labour and capital, and their relationship with output. Labour augmenting technical progress is in the *t* period. This relationship can best be explained in the equation below:

Let


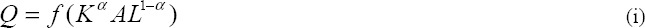


where Q is the total output produced in the given country; L is the total labour input used in the production process; K is the total capital input used in the production of Q; while A is the level of technology.

Therefore, equation (i) can be augmented by incorporating human capital (H), where H is the stock of human capital and can also be affected by other factors, like education and health, among others.


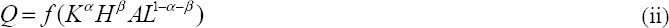


Equation (ii) can be transformed into a log-linear equation; hence, L represents the variable labour force productivity, K is the gross capital formation, and H represents the human capital.


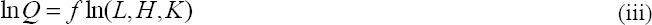


Other variables, such as the life expectancy rate, health, and education, can be used as a proxy for human capital to measure the quality of labour in the production process; introducing them into equation (iii) above, we have:


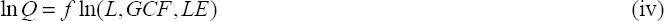


where L is the labour force productivity, GCF represents gross capital formation, and LE stands for the life expectancy rate.

Grossman (1972) viewed the health stock as an inherited stock possessed by labour, which depreciates over time through age, habit, accident, epidemics, and the like but can be improved by investment in health through exercise, diet, medical care services, having a good wife/husband, and so on. He differentiated human capital from health capital, in which human capital consists of the skills or knowledge acquired by labour through education and training, which can be expressed in market and non-market values, whereas health capital can be viewed as a durable capital stock that produces an output of healthy time. Therefore, equation (iv) above can also be transformed by incorporating health variables that can affect the stock of health, such as HIV/AIDS; hence, we have:


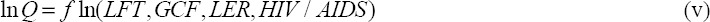


where HIV/AIDS stands for human immunodeficiency virus/acquired immune deficiency syndrome and the prevalence of HIV/AIDS in labour aged 14 to 45 years.

## 3. Literature Review

The African region remains the most heavily affected region in the global HIV/AIDS epidemic. In 2011, an estimated 23.5 million people living with the virus resided in Africa, which represents 60% of the global HIV burden (UNAIDS, 2012). Quinn (1996) reported the global burden of the HIV pandemic and noted that Africa and Asia were at the top of the list compared with the rest of the world. These findings were consistent with Corrigan et al. (2005), who investigated the relation between the AIDS crisis and growth in Sub-Saharan Africa using the general equilibrium model. They found that the macroeconomic consequences of the AIDS epidemic are quite large.

Different econometric methods and models have been employed by several authors and researchers to investigate the effect of the prevalence of HIV/AIDS on economic indicators, and the effect/impact of the former is not always in favour of the latter in the region. For example, Dixon et al. (2001) reported that the prevalence of the epidemic has a very large negative effect on life expectancy and economic growth in Africa. A two-least-square (2SLS) model was used by Garima Malik (2006) to examine the relationship between health and economic growth in India and the results indicated a negative relationship between HIV/AIDS and the GDP per capita growth rate in the country within the reporting period.

McDonald and Roberts (2006) employed an augmented Solow model, a human capital approach, to estimate the elasticity of AIDS in relation to economic growth, and the result revealed that HIV/AIDS has a negative impact on the income per capita in Africa, with an estimated coefficient of -0.59. This means that with a 1% increase in the epidemic (AIDS), the income per capita decreases by 0.59%.

The literature reviewed above indicated that the elasticity of the prevalence of HIV/AIDS is inelastic, implying that the percentage increase in HIV/AIDS is greater than the percentage decrease in economic growth due to the fact that the prevalence is increasing globally and diseases have no geographical boundary; as such, AIDS accounts for a greater fraction of the global distribution of communicable diseases. Africa is at the top of the list, with more than 16 million individual carriers of the disease, almost 3% of the population of the entire sub-continent (Quinn, 1996). However, this figure rose to 23.5 million people living with the virus in Africa, representing 60% of the global HIV burden (UNAIDS, 2012).

On the other hand, the effects of life expectancy on economic growth have gained much attention in the economic literature in recent years. Economic theory suggests that the increasing life expectancy induces individuals to obtain more schooling and hence affects their income positively. Acemoglu and Johnson (2006) examined the role of disease and development and the effect of life expectancy on economic growth. They found that an increase in life expectancy increases population growth, while an increase in life expectancy is significantly and positively related to economic growth. Again, Acemoglu and Johnson (2007) showed that an increase in life expectancy, instrumented by predicted mortality reductions, increases the population but has a weak and insignificant effect on growth. Using a general equilibrium model, Turan (2009) found a weak but positive relationship between life expectancy and economic development in Sub-Saharan Africa. A study by Hansen (2013), using a cross-country panel data analysis, found that a 1% rise in life expectancy at birth increases the years of schooling by 3.5%, which can influence income positively. Very few studies have investigated the relationship between the life expectancy rate and HIV/AIDS, including the work of Oster (2012), who studied the relation between HIV and sexual behaviour change in Sub-Saharan Africa and found a positive relationship between risky sex and HIV. Recently, Kunze (2014) used an overlapping generation model to investigate the relationship between life expectancy and economic growth and found that life expectancy affects growth positively by raising the savings rate, which eventually improves the future consumption.

## 4. Methodology

### 4.1 Model Specification

This research applied the panel data method of analysis to 33 Sub-Saharan African countries to measure the effect of the prevalence of HIV/AIDS and life expectancy on economic growth. The difference GMM, as proposed by Arellano and Bond (1991), was considered for the analysis because it is a dynamic model that allows for a dynamic relationship between the variables under consideration. Again, this method is important because the lagged value of the dependent variable is also part of the independent variables and this will help to capture the dynamic relationship. The data on capital flight were calculated based on the formula suggested by Schneider (2003). It is called the broad measure of capital flight and is defined as: CF = H + B + A + F, where *CF* is capital flight, H is the sum of the change in debt of a country; B is the net foreign direct investment; A is the current account surplus; and F is the change in reserves. Capital flight is introduced into the model as a variable to capture the fact that money that flees out of the economy affects growth negatively. The models are divided into two, with human immunodeficiency virus (HIV) and life expectancy (LE) considered as the dependent variables for each of the two models. This consideration is important to counteract the possible endogeneity problem between HIV and LE.

From equation (v) shown in section II, an econometric model can be written as:


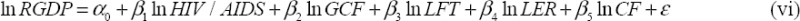


where RGDP is the percentage growth in the annual GDP per capita as the dependent variable; α_0_ is an intercept of the model; ln *HIV* / *AIDS* is the human immunodeficiency virus, which refers to the percentage of people aged between 15 and 49 years; gross capital formation, denoted by ln *GCF*, consists of additional fixed assets possessed by a given country, such as plant, machinery, and equipment purchases and the construction of road networks, active railway lines, and other physical structures, such as schools, hospitals, private residential dwellings, and commercial and industrial buildings; ln *LFT* is the labour force productivity, defined as the working population aged from 15 to 64 years; life expectancy is denoted by ln *LER*, defined as the number of years an individual would live from the date of his/her birth to the date of his/her death; ln *CF* is the capital flight; and ε is the residual of the model. Hence, the panel data model can be written as:


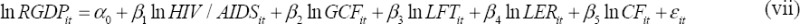


Equation (vii) can further be transformed into an empirical GMM model by estimating the equation and capturing the effect of the prevalence of HIV/AIDS and life expectancy on economic growth, while internally controlling for the possible endogeneity of the variables using moment conditions. This choice of modelling is informed by the fact that labour force productivity and life expectancy are most likely to be influenced by the prevalence of diseases such as HIV/AIDS; for example, a higher income is associated with a better health condition of the workers and sickness may influence their income negatively, thus reversing the causality (McKenzie & Sasin, 2007; Ngoma & Ismail, 2013; Ponce, Olivié, & Onofa, 2011).

Using the panel data set, the model is specified in two parts. Model 1 consists of HIV/AIDS without life expectancy, while the second model consists of life expectancy without HIV/AIDS to avoid the endogeneity problem. Model 1 is as follows:


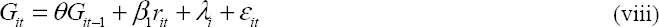


where *G_it_* represents the dependent variable of model 1, which refers to the log percentage of GDP per capita (growth rate) for country at time t; *θG*_*it*−1_ stands for the lag of the dependent variable; and *r_it_* is the vector of independent variables used in the research, such as HIV/AIDS, gross capital formation, labour force productivity, and capital flight. Meanwhile, *λ_i_* represents the unobserved country-specific effects, such as the geographical location and institutional factors, and *ε_it_* is the time-varying error term. Meanwhile, model 2 is specified as:


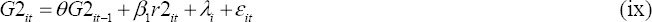


where *G2_it_* represents the dependent variable, which refers to the log percentage of GDP per capita (growth rate) for country *i* at time t; *θG*2_*it*−1_ stands for the lag of the dependent variable; and *r2_it_* is the vector of independent variables (without HIV/AIDS), such as the life expectancy rate, gross capital formation, labour force productivity, and capital flight. Meanwhile, *λ_i_* represents the unobserved country-specific effects, such as the geographical location and institutional factors, and *ε_it_* is the time-varying error term.

The models are estimated using the lagged difference dependent variables as an instrument to control the problem of autocorrelation of the first and second order and the lagged values of the explanatory variables as instruments to control for endogeneity in the regressors. Moreover, a two-step equation system is employed, which produces consistent estimates of the variance covariance matrix, which is robust to panel-specific heteroskedasticity and allows a robust test of instrument validity (Arellano & Bond, 1991; Ngoma & Ismail, 2013). Therefore, based on the theoretical prediction discussed in the literature review, we expect a negative coefficient of HIV/AIDS and capital flight. However, life expectancy, gross capital formation, and labour force productivity, respectively, could affect the economic growth of the region positively.

The elasticities of the two-step coefficients will be calculated by dividing the coefficient of the independent variables by one minus the coefficient of the lagged dependent variable, respectively, as follows:

Elasticity = *β*_0_ / 1−α_0_, where β_0_ is the coefficient two-step independent variable and α_0_ is the coefficient of the lagged dependent variable, respectively.

### 4.2 Variables and Data Source

The variables adopted for this study include the percentage growth in the annual GDP per capita as a proxy for economic growth as the dependent variable, while HIV/AIDS, life expectancy (LE), gross capital formation (GCF), labour force productivity (LFT), and capital flight (CF) are the independent variables, respectively. The data are generated from the World Development Indicators (World Bank database). Based on the availability of data, we selected the most recent possible sample of 33 countries out of 48 Sub-Saharan African countries for a period of 11 years (2002–2012).

Table 1 above presents the summary statistics of all the variables used in this study. The overall mean of the change in the annual GDP per capita growth is 6.521, which indicates that, on average, the GDP per capita of Sub-Saharan African countries grew by 6.521% annually within the reporting period. The physical capital denoted by gross capital formation on average grew by 3.087% per annum in the region, while the prevalence of HIV/AIDS amongst the population aged 14–64 years increased by 5.417% per annum. The labour force productivity, which is measured by the number of working population members aged between 15 and 64 years, on average increased by 14.864% per annum. The life expectancy rate measured at birth also increased annually on average by 4.009%, while capital flight increased by 22.543% per annum, respectively.

**Table 1:** Summaries of Statistics

Variables	Obs.	Mean	Standard Dev.	Min.	Max.
**GDP per Capita-Growth (% Annual).**	264	6.521	0.987	4.968	8.809
**Gross-Capital Formation(annual growth rate)**	264	3.087	0.392	1.698	4.374
**% Prevalence of HIV/AIDS (14-64years)**	264	5.417	7.188	0.2	27.4
**Labour-Force productivity (14-64 years)**	264	14.864	1.345	12.106	17.748
**Life-Expectancy (at birth) Capital flight**	264 264	4.009 22.543	0.120 1.546	3.741 18.139	4.311 26.476

To avoid the problem of endogeneity between our two hypothesis variables, that is, HIV/AIDS and the life expectancy rate, and to achieve more robust results, we divided them into two isolated models. The result is presented in Tables 2 and 3 below:

**Table 2:** 

Model	One step	Two Step

	Coefficient	Coefficient
θG_i,t-1_	0.907***(0.022)	0.919***(0.014)
HIV/AIDS	-0.015**(0.007)	-0.014***(0.002)
Gross Capital formation	0.018(0.015)	0.238*(0.007)
Labour force productivity	-0.387(0.253)	-0.563***(0.092)
Capital flight	-3.880(1.460)	-41.547(2.460)
**Abond test for auto correlation:**		
AR (1) [P-Value]	0.005	-
AR (2) [P-Value]	0.954	-
Sargan Test [P-Value]	-	41.469{0.003}
Cross section observations	33	33

*Note.* Values in parenthesis are standard errors

***, **, * represent 1%, 5% and 10% respectively, {} also indicate p-value for Sargan test for autocorrelation.

**Table 3:** Result of model 2 difference GMM estimations, Dependent variable: GDP growth rate (annual%)

Model	One step	Two Step

	Coefficient	Coefficient
θG2_i,t-1_	0.756***(0.049)	0.761***(0.031)
Gross Capital formation	0.016(0.014)	0.058**(0.007)
Labour force productivity	-0.184(0.247)	-1.0223***(0.074)
Life expectancy rate	0.983***(0.258)	0.395***(0.167)
Capital flight	-3.660(1.360)	-5.281(2.720)
**Abond test for auto correlation:**		
AR (1) [P-Value]	0.008	-
AR (2) [P-Value]	0.953	-
Sargan Test [P-Value]	-	39.89{0.005}
Cross section observations	33	33

*Note.* Values in parenthesis are standard errors.

***, **, * represent 1%, 5% and 10% respectively, {} also indicate p-value for Sargan test for autocorrelation.

Table 2 presents the results of one- and two-step Arellano and Bond dynamic panel data estimation as well as the Arellano–Bond and Sargan tests for autocorrelation and overidentification, respectively. The coefficient of the lagged dependent variable is positively related to the current GDP (growth) and significant at the 1% level in all the steps. In one step, HIV/AIDS shows a negative and significant coefficient, gross capital formation is positive but insignificant, while labour force productivity and capital flight have negative and insignificant coefficients, respectively. Therefore, there is a need to proceed to the two-step estimation to achieve a more robust result. The result of the two-step estimation reveals that HIV/AIDS is negatively correlated with the economic growth of the region, with a coefficient of -0.014 and significance at the 1% level. Regarding the other independent variables, gross capital formation is positively related to growth and significant at the 10% level, which is also consistent with economic theory; labour force productivity is negatively correlated with growth and significant at the 10% level; and capital flight has a negative but insignificant coefficient. The model passed the robustness test: in the Arellano–Bond test for autocorrelation, AR (1) [P-Value] is equal to 0.005 and AR (2) [P-Value] is equal to 0.954. The result is consistent with Arellano and Bond (1991). It also refers to the difference estimator, which proposes a two-step GMM estimator: One step in which the error terms are assumed to be independent and homoskedastic across countries and over time; and in Two step in which the residuals obtained in the first step are used to construct a consistent estimate of a variance–covariance matrix, thus relaxing the assumptions of independence and homoskedasticity that this step makes more efficient. Since this result passed the Sargan test for overidentifying restrictions (endogeneity), it can be suggested that the result is robust.

The coefficients of the lagged dependent variable, life expectancy, and gross capital formation produced a positive and significant result, whereas those of labour force productivity and capital flight indicated negative coefficients. Model 2 also passed the Arellano–Bond and Sargan tests for autocorrelation and over identification of restrictions, respectively.

### 4.3 Discussion

The elasticity of HIV/AIDS is inelastic such that the percentage increase in the prevalence of HIV/AIDS is greater than the percentage decrease in economic growth in Sub-Saharan Africa. This implies that a 10% increase in the prevalence of HIV/AIDS leads to a 0.14% decrease in the GDP of the region, and this finding is consistent with the theory and supported by previous studies (Asiedu et al., 2012; Gardner & Lee, 2010) in the case of the panel study. The other independent variables are all inelastic and produced mixed results. Gross capital formation is positively related to growth in both model 1 and model 2 and is consistent with the findings of Khanlen (2012) in the case of Nigeria. Labour force productivity is negatively related to growth, unlike the findings of Ammann (2013) and McNeill (2013) for Canada, where there is a positive relationship between labour productivity and economic growth.

Life expectancy has the highest elasticity of 0.98 among the independent variables and is positively related to growth. This finding implies that a 10% increase in life expectancy leads to a 9.8% increase in the GDP, and it is supported by many other studies, like Bhargava and Yu (1997) and Bloom et al. (2004). Bhargava et al. (2001) in fact suggested that an increase of 10% in the life expectancy at birth leads to a 0.3 to 0.4% increase in economic growth for the case of Africa.

## 5. Conclusion and Recommendation

This paper focused on the effect of the prevalence of HIV/AIDS and the life expectancy rate on economic growth in 33 Sub-Saharan African countries based on the availability of data over a period of 11 years. The generalised method of moments (GMM) was employed to achieve the objectives of the study. The result reveals that HIV/AIDS is statistically significant and negatively related to growth, consistent with theory and supported by many previous researchers (Asiedu, 2002, 2012). The life expectancy rate is also positively related to growth, as in the study by De and Licandro (1999), and consistent with economic theory. Labour force productivity is negatively related to growth, while gross capital formation is positively related, which is also consistent with economic theory.

Based on the findings of this research, the following recommendations are made. There is a need for future studies to determine the effect of HIV/AIDS and the life expectancy rate on economic growth in the region using a micro approach, because having contact with those involved would provide a true picture of their health status. Community effort in educating people, especially schoolchildren, about the danger of communicable diseases such as HIV/AIDS is strongly recommended. The family should spearhead the enlightenment of their wards from home to reduce or stop risky behaviours. Governments of SSA in partnership with NGOs should develop strategies for fighting the disease, such as making anti-retrovirus available and affordable in health service delivery centres, and funds generated from international donor agencies should be effectively and efficiently utilised in fighting HIV/AIDS and other related diseases in the region. The result of life expectancy shows a positive relationship with economic growth in the region. This is an indication that the factors influencing life expectancy, such as infant mortality, maternal health, and environmental factors, need to be given serious attention if sustainable development is to be achieved in the Sub-Saharan African region. Finally, the research was unable to capture social capital in its analysis as one of the important variables that determines the GDP and this can serve as a literature gap for future studies to fill.
